# CHIVA to spare the small and great saphenous veins after wrong-site surgery on a normal saphenous vein: a case report

**DOI:** 10.1590/1677-5449.007718

**Published:** 2019-01-07

**Authors:** Felipe Puricelli Faccini, Ani Loize Arendt, Raphael Quintana Pereira, Alexandre Roth de Oliveira

**Affiliations:** 1 Hospital Moinhos de Vento – HMV, Cirurgia Vascular, Porto Alegre, RS, Brasil.; 2 Hospital Moinhos de Vento – HMV, Cirurgia Cardiovascular, Porto Alegre, RS, Brasil.; 3 Hospital Moinhos de Vento – HMV, Anestesiologia, Porto Alegre, RS, Brasil.

**Keywords:** wrong-site surgery, CHIVA, venous operation, saphenous vein sparing, cirurgia em sítio errado, CHIVA, safena, preservação

## Abstract

CHIVA (Cure Conservatrice et Hemodynamique de l’Insufficience Veineuse en Ambulatoire) is a type of operation for varicose veins that avoids destroying the saphenous vein and collaterals. We report a case of CHIVA treatment of two saphenous veins to spare these veins. The patient previously had a normal great saphenous vein stripped in error in a wrong-site surgery, while two saphenous veins that did have reflux were not operated. The patient was symptomatic and we performed a CHIVA operation on the left great and right small saphenous veins. The postoperative period was uneventful and both aesthetic and clinical results were satisfactory. This case illustrates that saphenous-sparing procedures can play an important role in treatment of chronic venous insufficiency. Additionally, most safe surgery protocols do not adequately cover varicose veins operations. Routine use of duplex scanning by the surgical team could prevent problems related to the operation site.

## Resumo

*Cure conservatrice et hemodynamique de l’insufficience veineuse en ambulatoire* (CHIVA) é um tipo de cirurgia de varizes que evita a destruição da veia safena e colaterais. Este relato apresenta uma paciente que foi submetida a CHIVA em duas safenas para poupá-las. A paciente teve uma safena magna normal retirada em uma cirurgia no sítio cirúrgico errado, as safenas com refluxo foram mantidas, e uma normal foi ressecada. A paciente estava sintomática e foi realizada CHIVA na safena parva direita e na magna esquerda. O pós-operatório transcorreu bem com resultado clínico e estético satisfatório. Esse caso mostra que cirurgias que poupam a safena têm papel importante no tratamento da insuficiência venosa crônica. Além disso, os protocolos de cirurgia segura não cobrem adequadamente as cirurgias de varizes devido a duas safenas possíveis e por serem frequentemente cirurgias bilaterais. A realização de eco-Doppler rotineiramente pela equipe cirúrgica pode prevenir problemas relacionados ao sítio operatório.

## INTRODUCTION

 CHIVA is the French acronym for “Cure conservatrice et Hemodynamique de l’Insuffisance Veineuse en Ambulatoire” (Conservative and Hemodynamic treatment of the Venous Insufficiency in the office). It is a saphenous-sparing therapeutic approach for lower limb chronic venous disease (CVD) based on hemodynamic concepts proposed by Claude Franceschi in 1988. 1 The rationale behind this hemodynamic approach to treat the disease is that it is increased transmural pressure (TMP) that is responsible for the progression of the signs and symptoms of CVD, such as varicosities, edema, pain, itching, dermatitis and ulcers. In superficial venous disease, TMP is increased because of higher hydrodynamic pressure caused by the absence of orthodynamic pressure fractionating and the presence of closed shunt (deep superficial). 

## CASE REPORT

 A 46-year-old female presented in 2017 with symptomatic right leg pain and aesthetic complaints relating to the right calf. Medical history showed a previous head trauma (car accident) with brain hematoma drainage and a saphenous vein operation. Physical examination revealed edema in the perimalleolar area and painful varicose veins, in the right calf (with considerable aesthetic impact) and left calf (with minor aesthetic impact). Venous scores at the first visit to our clinic were the following: Venous clinical severity score VCSS 10 and Aberdeen quality of life questionnaire 27.7. 

 Duplex examination conducted before the original venous operation (which had been performed in a different clinic in January 2016) had shown reflux in the left great saphenous vein and significant reflux in the right small saphenous vein. However, the operation actually performed was stripping of the right great saphenous vein. Both the left great saphenous vein and the right small saphenous veins were left in place untreated. After this procedure, symptoms had exacerbated progressively, and the aesthetics of the leg had deteriorated progressively. 

 Preoperative evaluation was normal. We performed a complete duplex scan, according to our routine, as published elsewhere. 2 The patient had type 1b+2a shunt in the right leg and 4+2d shunt in the left leg. We suggested operating to treat the small saphenous vein in the right leg and the great saphenous vein in the left leg. We treated the patient using the CHIVA technique to preserve the remaining saphenous veins. 

 We performed the CHIVA procedure on both legs during the same operation. Local anesthesia was provided with a solution containing 10 mg/mL 20 mL of ropivacaine and 2% lidocaine, using 20 mL and 60 mL of saline. We routinely have an anesthetist in the operating room to guarantee patient safety and comfort, who is always advised to avoid sedation as much as possible. When necessary, an opioid-free sedation technique is employed. 3 In the right leg, we ligated the small saphenous vein at its junction with a calf vein and ligated two N3 collaterals, leaving the small saphenous vein draining through two perforators. In the left leg, we ligated a collateral draining to the great saphenous vein from the inguinal ligament and an N3 draining reflux from the great saphenous vein to the calf. A total of 5 small incisions were made. The patient was discharged two hours after the operation wearing compressive stockings and taking 40 mg enoxaparin per day for 3 days, according to our postoperative routine. 

**Figure 1:**
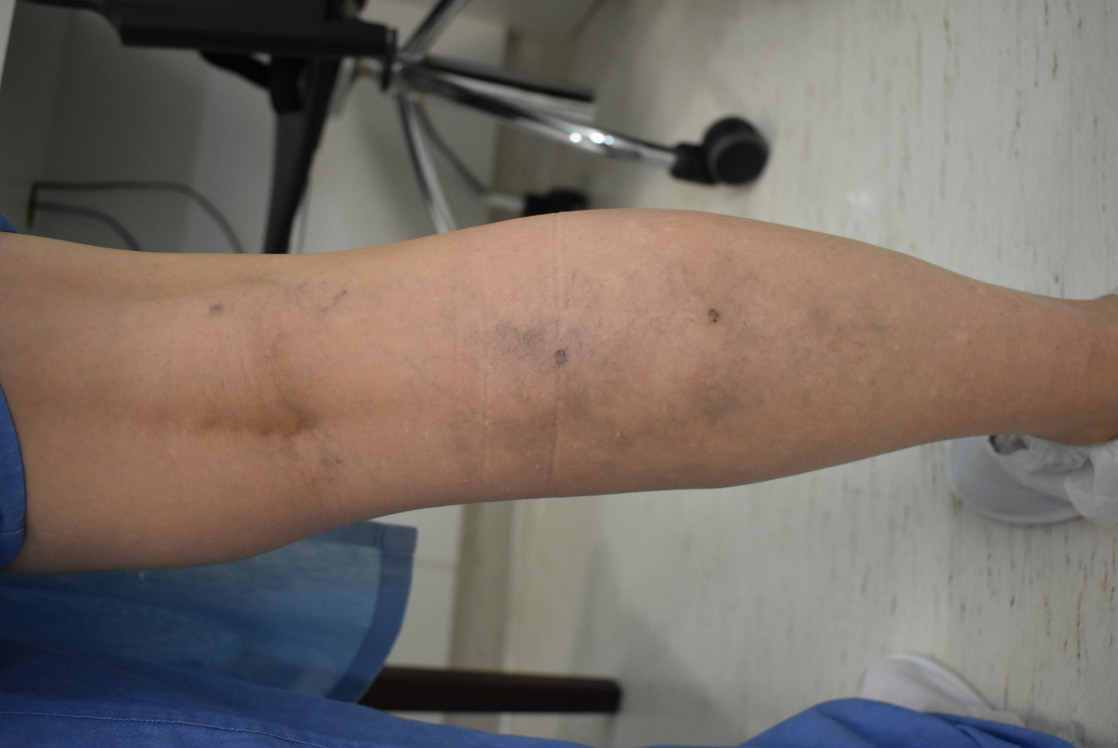
Preoperative photograph of the right calf.

**Figure 2:**
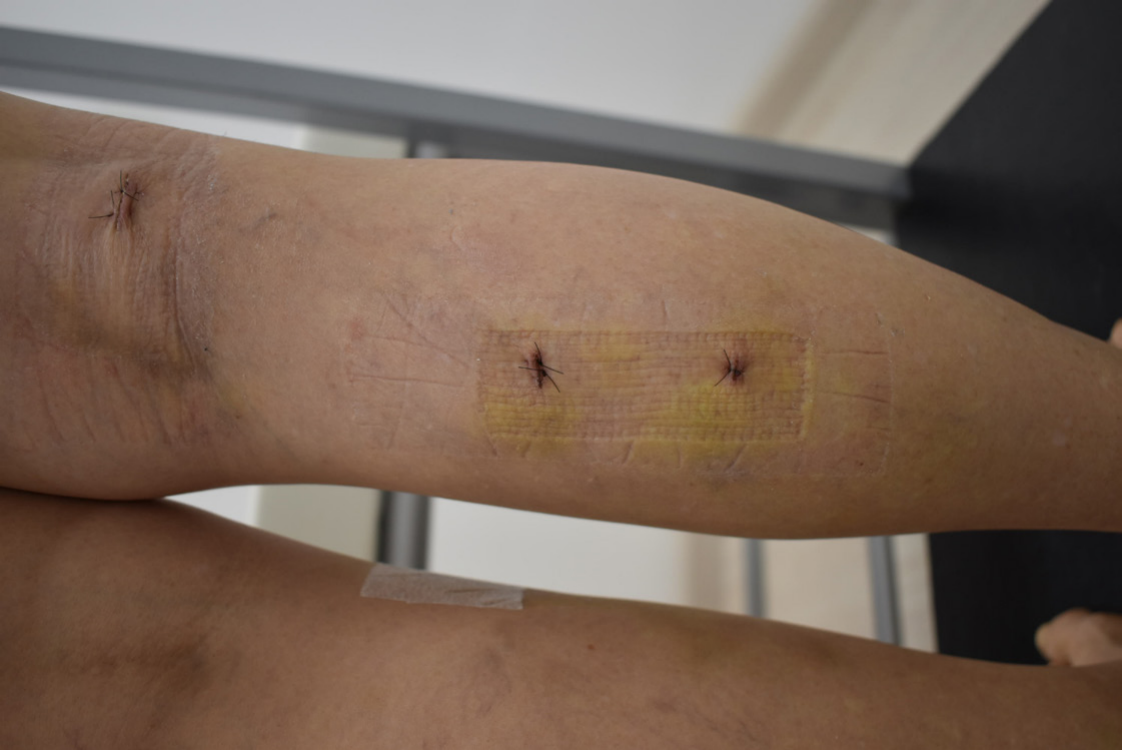
Postoperative photograph of the right calf.

 On the sixth postoperative day, duplex scanning was performed, showing minor continuous reflux in the small saphenous vein of the right leg and even less reflux in the great saphenous vein on the left. The right small saphenous vein had been 7.4 mm before the operation and was 3.8 mm after. The left great saphenous vein had been 4 mm before the operation and had not decreased in size during the initial postoperative period. The patient scored pain at 3 on a 0-10 pain scale and had taken one 750 mg paracetamol tablet during the entire postoperative period. We made a full photographic record before and after the operation ( Figures 1 and 2 ). There were no photographs or records of symptoms available from the original operation. 

 In relation to the wrong-site surgery, we comforted the patient and reported the case to both the previous surgeon and the patient safety surveillance team at the hospital where the operation had been performed. 

## DISCUSSION

 We are calling attention to this case because of two important aspects; the need for continuous focus on safe surgery and the importance of saphenous vein sparing operations (pivotal in this case, after a normal saphenous vein has been removed). 

 There are several possible procedures for treatment of varicose veins that achieve good results. The CHIVA strategy is based on the hemodynamics of the venous system and aims to maintain the venous system in place, while correcting imbalances created by shunts between the deep and superficial venous systems. 1 The main characteristics of the procedure are: a: local anesthesia, b: day-clinic surgery, c: immediate return to activities, d: low pain scores, e: avoidance of removal of collaterals, causing fewer skin blemishes, and f: saphenous veins are left in place for future use in bypasses. In the case presented here, preservation of the saphenous vein was not only important for future bypass and recurrence “guidance”, but, additionally, the patient desired to maintain the veins and have the procedure under local anesthesia. The fact that the great saphenous vein on the right had been stripped in a wrong-site surgery prompted the patient to request not to be sedated in order to remain in control of the situation. 

 The importance of preservation of the saphenous veins has been gaining ground over recent years 4 and aspects of venous operations are the subject of great debate. A thorough discussion is beyond the scope of this report. Possible advantages of preservation include keeping the vein for further use in bypass surgeries, reducing surgical trauma to prevent remodeling, and maintaining the saphenous trunk to receive flow in case of a recurrence. Biochemical studies suggest that an increase in the pressure on veins and chronic shear stress of the vein wall are linked to venous remodeling and may lead to recurrence. 5 ^,^ 6 Animal studies have shown that transcription factor activator protein 1 (AP–1) appears to be a prerequisite for venous remodeling/proliferation and MMP–2 [matrix metalloproteinases] expression. MMP-2 expression is stimulated by sudden interruption of the ear vein in rats. Clinical studies support the view that ligation of all junctional saphenous tributaries is associated with a higher risk of varicose vein recurrence. 7 These data suggest that an approach with less resection may help reduce recurrence. 

 Concerning the results and safety of CHIVA, a recent Cochrane systematic review including clinical trials evaluating CHIVA compared to stripping showed less nerve damage, fewer bruises, and less recurrence. 8 The results favored the CHIVA approach, although the review authors suggested further studies are necessary to corroborate findings and further evaluate results with quality of life questionnaires. 8 Two previously published randomized clinical trials comparing stripping and CHIVA evidenced no nerve damage in 286 CHIVA procedures and 26 nerve damage events in 383 (6.7%) stripping procedures. 9 ^,^ 10 During a CHIVA procedure under local anesthesia, the patient alerts the surgeon if the sural or saphenous nerves are touched, which does not happen with general, axial, or tumescent anesthesia because the nerve or response are blocked, thereby leaving the nerve susceptible to damage by the mechanical or thermal energy used in most procedures. Pares et al. 10 reported that 240 out of 334 patients (71%) presented bruising after stripping compared to 76 out of 167 (45%) patients in the CHIVA group. This happens because most veins are left in place and less blood is left in the subcutaneous area to stain the skin. Recurrence of disease was also evaluated in clinical trials after 5-10 years follow-up. 9 ^-^ 11 These clinical trials showed recurrence in 81 patients out of 286 (28%) in the CHIVA group and 205 out of 435 patients (47%) in the stripping group. Additionally, Zamboni et al. published a trial comparing venous ulcer healing in patients after CHIVA or compression treatment, showing that CHIVA results are consistent even in ulcer cases. 12 The study showed that the recurrence of venous ulcers during a 3-year follow-up was significantly lower in the CHIVA group (9%) compared to compression group (38%). 

 After any case of wrong-site surgery, a discussion of what should be done to avoid it is imperative. Accordingly to Hanchanale et al., 13 the main factors contributing to wrong operations are environmental distractions, team fatigue, multi-surgeon teams, lack of communication between team members, confusing exam reports and lack of adherence to a safe surgery protocol. These authors suggest that strictly following the safe surgery protocol and investigating cases (or near-miss cases) to improve the protocol are the mainstay to avoid these events. 13 We reported the case to the surgeon responsible for the previous operation and discussed the protocols at our hospital to evaluate their adequacy. From the hospital perspective, we concluded that the checklist and the “TIME OUT” of surgical protocols are important and complete for most operations. However, it might miss some cases because the term bilateral is commonly used at the checkpoint of the safety protocol in varicose veins surgery. This term is confusing for some operations and chronic venous disease cases are particularly problematic. Patients have four saphenous veins to be treated or not during a bilateral procedure for venous disease. In the majority of these procedures, only one is operated and the others are left untreated. We decided to add a question to the checklist about which saphenous veins should be treated. The protocol now asks for side during checklist (right, left, or bilateral) and if the answer is ‘bilateral’ the surgeon should specify the veins to be treated. This may reduce such occurrences and is highly important in hospitals with several teams and surgeons working independently, in which team safety measures are more difficult to implement. 

 From the vascular surgeon’s perspective, safety in venous operations depends on who performs the duplex scan and when it is performed. In our team, a duplex scan is always performed by the surgeon responsible for the patient on the same day as the operation, while marking the surgical sites on the skin. Unfortunately, there is pressure from insurance companies to have the duplex scan conducted prior to the operation for auditing purposes and in some cases there is no reimbursement for a second ultrasound examination on the day of the operation. Additionally, the daily routine of surgical teams can make it difficult for surgeons to perform same-day duplex scans in all cases. Some surgical teams have several members and not all are trained in duplex scanning, which makes same-day examination more difficult. In the case reported in this paper, we are confident that a same-day duplex scan would have averted the wrong-site surgery. 
